# Steroid Hormone Control of Cell Death and Cell Survival: Molecular Insights Using RNAi

**DOI:** 10.1371/journal.pgen.1000379

**Published:** 2009-02-13

**Authors:** Suganthi Chittaranjan, Melissa McConechy, Ying-Chen Claire Hou, J. Douglas Freeman, Lindsay DeVorkin, Sharon M. Gorski

**Affiliations:** 1 The Genome Sciences Centre, BC Cancer Agency, Vancouver, British Columbia, Canada; 2 Department of Molecular Biology and Biochemistry, Simon Fraser University, Burnaby, British Columbia, Canada; University of California San Francisco, United States of America

## Abstract

The insect steroid hormone ecdysone triggers programmed cell death of obsolete larval tissues during metamorphosis and provides a model system for understanding steroid hormone control of cell death and cell survival. Previous genome-wide expression studies of *Drosophila* larval salivary glands resulted in the identification of many genes associated with ecdysone-induced cell death and cell survival, but functional verification was lacking. In this study, we test functionally 460 of these genes using RNA interference in ecdysone-treated *Drosophila l(2)mbn* cells. Cell viability, cell morphology, cell proliferation, and apoptosis assays confirmed the effects of known genes and additionally resulted in the identification of six new pro-death related genes, including sorting nexin-like gene *SH3PX1* and Sox box protein *Sox14*, and 18 new pro-survival genes. Identified genes were further characterized to determine their ecdysone dependency and potential function in cell death regulation. We found that the pro-survival function of five genes (*Ras85D*, *Cp1*, *CG13784*, *CG32016*, and *CG33087*), was dependent on ecdysone signaling. The TUNEL assay revealed an additional two genes (*Kap-α3* and *Smr*) with an ecdysone-dependent cell survival function that was associated with reduced cell death. *In vitro*, Sox14 RNAi reduced the percentage of TUNEL-positive *l(2)mbn* cells (*p*<0.05) following ecdysone treatment, and Sox14 overexpression was sufficient to induce apoptosis. *In vivo* analyses of *Sox14-RNAi* animals revealed multiple phenotypes characteristic of aberrant or reduced ecdysone signaling, including defects in larval midgut and salivary gland destruction. These studies identify Sox14 as a positive regulator of ecdysone-mediated cell death and provide new insights into the molecular mechanisms underlying the ecdysone signaling network governing cell death and cell survival.

## Author Summary

Hormones regulate complex signaling pathways required for the differentiation, growth, survival, and death of cells in diverse organisms. The insect steroid hormone 20-hydroxy ecdysone (ecdysone) triggers cell death of obsolete larval tissues, such as the midgut and salivary glands, during metamorphosis and provides a model system for understanding steroid hormone control of cell death and cell survival. Previous studies identified many genes and proteins associated with fruit fly salivary gland cell death, but functional verification was lacking. Here, we have analyzed 460 of those genes using RNAi, a genetic approach to inhibit gene function, to assess their possible cell death or cell survival related function. To our knowledge, this is the first large-scale functional screen for genes involved in steroid hormone regulated cell death and cell survival. We identified several novel ecdysone regulated components with a cell death/survival role, including genes with no previously known function. *In vivo* analyses of animals harboring an RNAi construct targeting the transcription factor *Sox14*, one of the genes identified, confirmed its role as a positive regulator of ecdysone-mediated cell death. Our results provide a foundation for further studies of the molecular mechanisms by which steroid hormones control the death and survival of cells.

## Introduction

Steroid hormones are small hydrophobic signaling molecules which bind to their receptors to control gene expression and initiate the regulation of growth, development, homeostasis and programmed cell death (PCD) [1]. Components of the steroid-regulated PCD transcriptional regulatory cascades in insects and mammals have been well characterized. For example, in vertebrates, steroid hormone glucocorticoids regulate the removal of excess thymocytes during T-cell maturation [2],[3]. In insects, the transcriptional cascade induced by the steroid hormone 20-hydroxyecdysone (ecdysone) has been implicated in the activation of PCD in larval intersegmental muscle [4]–[6], newly eclosed adult central nervous system [7],[8], larval salivary glands [9], and larval midgut [10]. Deregulation of the hormonal control of PCD in humans has been associated with various pathological conditions, including cancer and the degenerative disorder Alzheimer's Disease [1],[11],[12]. Given the functional conservation of many genes in humans and *Drosophila*, experiments to identify the genes required for hormonal control of *Drosophila* PCD will provide not only a better molecular understanding of the process itself, but may also be valuable in the context of human disease treatment and diagnostics.

During metamorphosis of *Drosophila*, two stage-specific sequential pulses of ecdysone activate first the transformation of larvae into pupae, and then the transformation of pupae into adult flies. The ecdysone pulses regulate the destruction of obsolete larval tissues, and the differentiation and morphogenesis of adult tissues which arise from small clusters of progenitor cells, [7], [8], [13]–[16]. The first ecdysone pulse occurs at the late third instar larval stage and triggers puparium formation. In addition, the larval midgut undergoes histolysis and the future adult midgut tissue envelopes it by 2 hrs after puparium formation (APF) [9],[10]. A second ecdysone pulse occurs 10 hrs APF and triggers the death of larval salivary glands [9]. Previous studies indicated that larval midguts and salivary glands employ similar, yet distinct, genetic mechanisms during steroid induced programmed cell death [10].

Several studies have identified some of the components involved in the transcriptional cascade upstream of PCD of salivary glands in *Drosophila*. The ecdysone receptor is a heterodimer of the nuclear receptors ecdysone receptor (EcR) and ultraspiracle (USP) [17]. The heterodimer complex binds to the steroid hormone ecdysone and induces the transcription of the early genes *E93* (DNA binding protein), *BR-C* (zinc finger transcription factor), *E74* (ETS-domain transcription factor), and *E75* (orphan nuclear receptor) [13], [18]–[22]. The EcR∶USP complex and E93, BR-C and E74 proteins in turn activate transcription of several pro-death genes including *reaper* (*rpr*), *head involution defective* (*hid*) and *grim* which function similarly to mammalian *SMAC/DIABLO*, the *APAF-1* homologue a*rk*, the initiator caspase *dronc*, and the CD36 receptor homologue c*roquemort* (*crq*) [14], [21], [23]–[30]. The transcription factor *E75B* is sufficient to repress transcription of the inhibitor of apoptosis protein 2 gene, *diap2* [21], while EcR and the CREB binding protein (CBP) transcriptional cofactor are required for *diap1* downregulation [31]. Functional studies have confirmed that at least *Ecr*, *E93*, *BR-C*, *hid*, *ark*, *dronc*, *CBP* (CREB binding protein), and *AP-1* (heterodimer of c-Jun and c-Fos) are required for *Drosophila* salivary gland cell death [9], [22], [27], [31]–[37]. Similarly, functional studies have identified a role for *Ecr*, *BR-C*, *E93*, *rpr and hid* in larval midgut cell death [10],[26],[38],[39]. While the upstream ecdysone signaling cascade and some cell death genes thus have a demonstrated function in these death processes, the results of genome-scale expression studies [23],[24],[40] suggest that there are many more potential effectors of ecdysone-regulated cell death and cell survival.

The *Drosophila* cell line *l(2)mbn* [41], a tumorous haemocyte cell line, is well suited for studying steroid hormone induced programmed cell death for several reasons. First, treatment of *l(2)mbn* (also known as mbn2) cells with ecdysone was shown to induce cell death with morphological features of apoptosis (DNA fragmentation, apoptotic bodies) and autophagy [42]. Second, ecdysone treatment induced the expression of the transcription factor *BR-C* and the caspases Dronc and Drice [43],[44] in *l(2)mbn* cells. And, third, following ecdysone treatment, the knock-down of *E93*, *BR-C* and caspases by RNA interference (RNAi) reduced *l(2)mbn* cell death [43],[44], while the knockdown of *E74B*, *E75A*, and *E75B* by RNAi enhanced cell death [44]. These features indicate that ecdysone mediated cell death in *l(2)mbn* cells is akin, at least in part, to dying larval stage *Drosophila* salivary glands and midgut.

Treatment of cultured *Drosophila* cells with double stranded ribonucleic acid (dsRNA) targeting specific genes depletes their corresponding transcripts and has been used as an efficient tool for genome wide loss-of- function phenotypic analyses [45]–[54]. Recent microarray, SAGE and proteomics studies [23],[24],[40] have identified hundreds of transcripts and proteins that are differentially regulated in *Drosophila* larval salivary glands immediately prior to ecdysone-induced cell death, but their functions in this process remain untested. Here we analyze the function of 460 of these gene products using RNAi in ecdysone treated *l(2)mbn* cells, and report the identification of many novel players in the ecdysone signaling network governing cell death and cell survival.

## Results

### Characterization of Ecdysone-Induced *l(2)mbn* Cell Death

**Figure 1:**
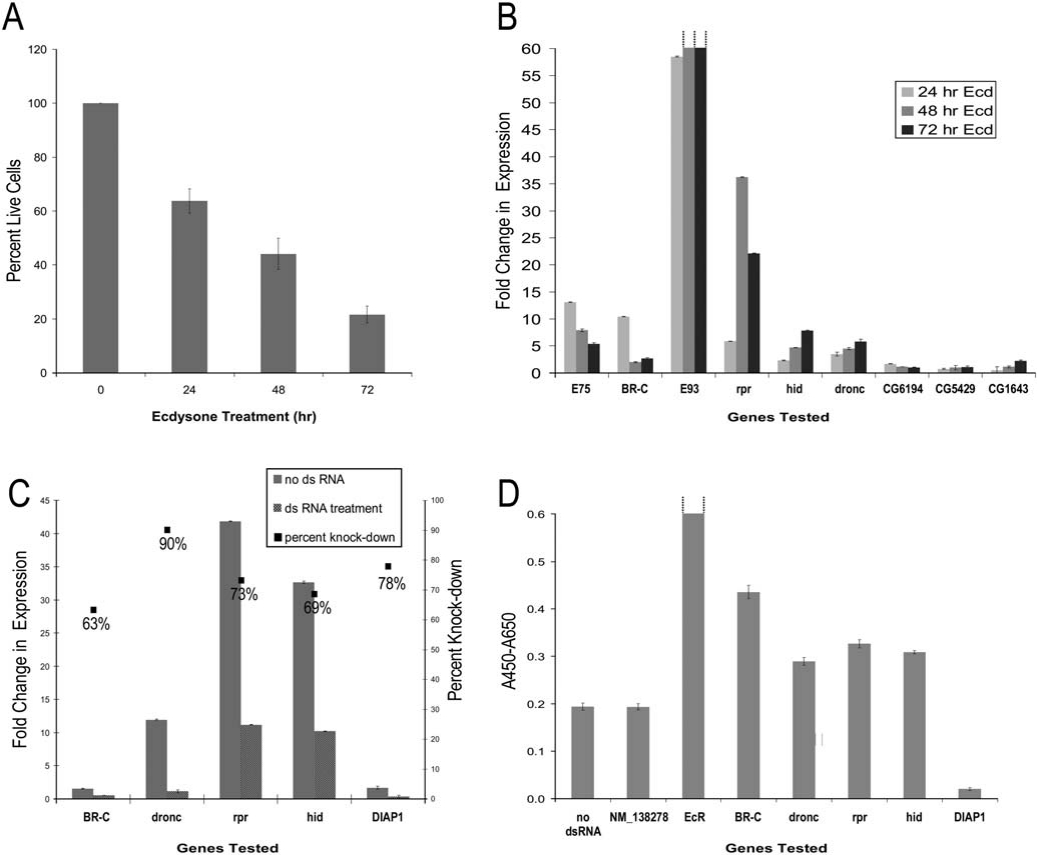
Ecdysone signaling and apoptosis genes are differentially expressed and required for cell death in ecdysone treated *l(2)mbn* cells. (A) *l(2)mbn* cells treated with 10 µM ecdysone showed a >70% reduction in live cells after 72 hours. Cells were exposed to 10 µM ecdysone for the times indicated. Surviving cells were counted by Trypan blue exclusion and percent live cells were calculated by comparing ecdysone-treated cells with untreated cells (100%). (B) QRT-PCR expression profiling showed that the ecdysone induced genes *E75*, *BR-C*, *E93*, *rpr*, *hid* and *dronc* had elevated levels of expression (at least 4 fold increase) in ecdysone treated (10 µM) *l(2)mbn* cells relative to untreated control *l(2)mbn* cells. The dotted lines above the bars for E93 indicate that the fold change in expression exceeded the existing scale (48 hrs, 126 fold change; 72 hrs, 371 fold change). The autophagy genes, *DmAtg4-like (CG6194)*, *DmAtg6(CG5429)*, and *DmAtg5(CG1643)*, did not show differential expression following ecdysone treatment. (C) QRT-PCR analysis of gene transcripts following treatment with the indicated dsRNAs and ecdysone. As shown here, the knockdown ranged between 63% and 90% for the representative gene transcripts tested following 72 hr dsRNA and ecdysone treatment compared to ecdysone treatment alone. (D) Cells treated with dsRNA corresponding to *BR-C*, *EcR*, *dronc*, *rpr*, and *hid* showed significantly (p≤0.05) increased levels of cell viability and those treated with dsRNA corresponding to *diap-1* showed significantly (p≤0.05) reduced levels of cell viability compared to cells treated with ecdysone and human dsRNA NM_138278 (negative control). Cell viability was measured by the WST-1 assay (A_450_–A_650_). The dotted lines above the bar for EcR indicate the A_450_–A_650_ value ( = 1.0) exceeded the existing scale. For (A)–(D), the error bars represent the SD of triplicate samples.

To validate our experimental system, we conducted cell viability, cell death, transcription and RNAi assays in ecdysone-treated *l(2)mbn* cells using known ecdysone signaling and apoptosis genes. First, to verify previous findings [42]–[44] of ecdysone treatment effects on *Drosophila l(2)mbn* cells, we employed multiple assays over a time course of ecdysone treatment. To assess cell viability, we used the trypan blue exclusion [55] and 4-[3-(4-iodophenyl)-2-(4-nitrophenyl)-2H-5-tetrazolio]-1,3-benzene disulfonate (WST-1) based cell viability (Roche Diagnostics) assays. Both assays indicated that the majority of cells are non-viable by 72 hours following treatment with 10 uM ecdysone (Figure 1A and 1D). To specifically measure cell death, nuclei were stained with DAPI and the percent TUNEL positive cells were determined 72 hours following ecdysone treatment. Our results showed that the control and ecdysone treated cells had 11% and 54% TUNEL positive cells, respectively, indicating that the reduced cell viability is due, at least in part, to increased cell death. In addition, we used electron microscopy (EM) to examine morphological features of *l(2)mbn* cells following ecdysone treatment. Consistent with previous reports [42], we observed features representative of apoptosis, autophagy and phagocytosis in the ecdysone treated cells (data not shown).

To determine the expression profile of representative ecdysone regulated transcription factors and apoptosis genes in *l(2)mbn* cells, we employed quantitative reverse transcription PCR (QRT-PCR) and measured transcript levels following 24, 48 and 72 hrs ecdysone treatment. Since we observed features of autophagy after ecdysone treatment, we also quantitated the expression levels of several autophagy genes to determine if their expression was ecdysone regulated in our experimental system. Our QRT-PCR results (Figure 1B) indicate that the early transcription factors *Br-C* and *E75* had the relatively highest expression levels at 24 hrs (10 and 13 fold increase in expression, respectively, compared to untreated control cells) and then decreased after 48–72 hours (at 48 hours, 2 and 7.9 fold increase in expression, respectively, compared to control cells). As demonstrated in Figure 1B, *E* *93*, *reaper*, *dronc* and *hid* demonstrated elevated expression levels by 24 hrs (58, 5.8, 3.4, and 2.3 fold increase respectively) which remained elevated or continued to increase at 48 and 72 hours (at 48 hours, 126, 36, 4.4 and 4.7 fold increase in expression, respectively). These observations suggest that the transcriptional cascade for the representative ecdysone signaling and apoptosis genes is similar between ecdysone-treated *l(2)mbn* cells and dying *Drosophila* larval salivary glands. Although we detected expression of autophagy genes in *l(2)mbn* cells, we observed no significant differential expression compared to untreated cells (ie. below the arbitrarily chosen 2 fold cut-off level) (Figure 1B) up to 72 hrs following ecdysone treatment, indicating that the autophagy genes tested are not transcriptionally regulated in this system at these timepoints (Figure 1B).

To test the sensitivity of our RNAi strategy, we treated *l(2)mbn* cells with dsRNA corresponding to representative ecdysone signaling (*EcR*, *BR-C* and *E75*) and apoptosis (*dronc*, *rpr*, *hid* and *diap-1*) related genes. First, to determine the knock-down efficiency of RNAi for the genes described above, we measured their expression levels at 72 hrs by QRT-PCR in ecdysone-treated cells with or without dsRNA. For all the genes tested, the transcript knock-down ranged between 62–90% (for examples, see Figure 1C). Next, the WST-1 assay was used to measure cell viability following RNAi and ecdysone treatment. We found that treatment of *l(2)mbn* cells with ecdysone and dsRNAs corresponding to *EcR*, *BR-C*, *dronc*, *reaper* and *hid* resulted in increased cell viability (p≤0.05) compared to cells treated with a negative control, a human dsRNA NM_138278 [56] (Figure 1D; Table 1). Treatment of *l(2)mbn* cells with dsRNA corresponding to either *E75B* (Table 1) or *diap-1* (Figure 1D; Table 1) decreased cell viability significantly (p≤0.001) as assayed by WST-1. We confirmed that the change in viability of the *l(2)mbn* cells treated with ecdysone and RNAi was due to alterations in cell death by employing the TUNEL and DAPI assay for selected genes (Table 2).

**Table 1:** Candidate pro-death and pro-survival genes identified by RNAi and cell viability assay.

Symbol or CG number of gene targeted	WST-1 assay: dsRNA+ecdysone	WST-1 assay: dsRNA only	Ecdysone dependency	predicted/known function	HGNC Symbol: Ortholog in human (mouse)
**Control pro-death genes**					
EcR	7E-05	***2E-02***	dependent	ecdysone receptor, transcription factor	NR1H3
Hid	3E-03	1E-01	dependent	apoptosis	
BR-C	2E-02	3E-01	dependent	transcription factor activity	ZBT12
dronc (Nc)	2E-02	4E-01	dependent	caspase activity	
**Ecdysone dependent candidate pro-death genes**					
RpL13A ^a^	6E-05	***2E-03***	dependent	60S ribosomal protein L13a	XR_000922.1
Sox14	7E-04	1E-01	dependent	transcription factor	Sox 4,11,22
RpS6 ^b^	2E-03	***3E-02***	dependent	40S ribosomal protein S6	RPS6
RpLP1 ^a^	3E-03	***3E-03***	dependent	60S acidic ribosomal protein P1	RPLP1
RpS5	6E-03	***2E-03***	dependent	40S ribosomal protein S5	RPS5
SH3PX1 ^b^	2E-02	5E-03	independent	intracellular protein transport	SNX9
RpL37	5E-02	***4E-05***	dependent	60S ribosomal protein L37	Rpl37
**Control pro-survival genes**					
th(diap-1) ^a^	9E-04	1E-05	independent	anti-apoptosis	
E75	7E-04	3E-01	independent	DNA binding; steroid hormone receptor	NR1D1
**Pro-survival genes (ecdysone dependent and independent)**					
sin3A	3E-04	1E-02	independent	transcription factor	SIN3B
S6K ^c^	6E-04	3E-03	independent	positive regulator of cell growth	RPS6KB1
Rpn2 ^a^	8E-04	6E-04	independent	endopeptidase	PSMD1; RYR2
Pros26.4 ^a^	9E-04	9E-04	independent	endopeptidase	PSMC1
Ras85D	2E-03	2E-01	dependent	G-coupled signaling, anti-apoptotic	HRAS
Smr ^a^	2E-03	4E-03	independent	DNA binding; protein binding	NCOR1
Vps32 ^a^	3E-03	6E-03	independent	carrier activity	CHMP4B
Tbp-1 ^a^	4E-03	9E-04	independent	endopeptidase	PSMC3
Tor ^c^	6E-03	6E-03	independent	protein kinase, cell growth, autophagy	FRAP1, mTOR
CG33087	7E-03	4E-01	dependent	Ca^++^ ion binding; ATPase, LDL receptor activity	LRP1
Indy	7E-03	7E-03	independent	tricarboxylic acid transporter	SLC13A2
Kap-α3 ^a^	7E-03	3E-05	independent	protein carrier	KPNA3
CG7466	9E-03	7E-03	independent	receptor binding; cell-cell adhesion	MEGF8
Cp1	1E-02	3E-01	dependent	cathepsin L activity; proteolysis	CTSL
CG32016	2E-02	8E-02	dependent	unknown	
HmgD^a^	2E-02	3E-03	independent	DNA binding activity	
CG13784	2E-02	7E-01	dependent	unknown	
CG15239	4E-02	1E-03	independent	unknown	

Gene symbols, CG numbers (column 1), and functions (column 5) are from Flybase [58]. The indicated Ortholog symbols (column 6) are from the HUGO Gene Nomenclature Committee (HGNC). P-values in columns 2 and 3 were calculated by comparing the WST-1 reading (A450–A650) from RNAi of the gene of interest to the WST-1 reading from RNAi of the human (Hs) negative control (NM_138278). Ecdysone dependent means that the observed viability effects of RNAi depended on the presence of ecdysone. Italicized and bolded P-values in column 3 indicate that these genes showed a pro-survival effect in the absence of ecdysone. Each RNAi treatment had three replicates and the assay was conducted at least twice. Superscript symbols a, b, and c indicate that these genes were identified in other related RNAi screens; a = Boutrous et al., 2004 [49]; b = Björklund et al., 2006 [47]; c = Bettencourt Dias et al., 2004 [57].

**Table 2:** Ecdysone dependent and ecdysone independent death-related effects identified by the TUNEL assay.

Gene Targeted	Ecdysone treatment			No ecdysone treatment		
	Percent dead cells *	TUNEL Assay	p-value	Percent dead cells *	TUNEL Assay	p-value
No Ecd or dsRNA treatment				11		
Ecdysone treatment	54					
NM_138278	56	control		9	control	
**Control Pro-death genes**						
BR-C	13	− −	4E-05			
dronc (Nc)	24	−	3E-02			
**Control Pro-survival genes**						
th (diap-1)	76.8	+ +	4E-06			
**Candidate Pro-death Genes**						
Hid	13	− −	3E-05			
RpLP1	34	− −	1E-04			
RpL13A	38	− −	3E-04			
EcR	9	− −	5E-03			
RpL37	29	− −	5E-03			
RpS5	44	− −	8E-03			
Sox14	37	−	3E-02			
SH3PX1	47	−	3E-02			
RpS6	54	NC	5E-01			
**Pro-survival Genes**						
**Group A: Increased TUNEL positive cells only in the presence of ecdysone**						
Kap-α3	85	++	4E-05	14	NC	1E-01
CG32016	72	++	6E-03	21	NC	5E-02
Ras85D	65	++	1E-02	11	NC	4E-01
Smr	69	+	2E-02	17	NC	1E-01
**Group B: Increased TUNEL positive cells in the absence or presence of ecdysone**						
S6K	72	+	2E-02	21	+	5E-02
Pros26.4	65	+	4E-02	31	++	3E-04
**Group C: Increased TUNEL positive cells only in the absence of ecdysone**						
Tor	62	NC	5E-02	23	++	2E-03
sin3A	64	NC	1E-01	20	+	3E-02

Gene names are from Flybase [58].

*: Percent dead cells = Number of TUNEL positive cells/Number of DAPI positive cells ×100. P values were calculated by comparing the percent dead cells of each RNAi treatment to the human control (NM_138278) RNAi treatment (++ = significantly increased TUNEL with p≤0.01; + = significantly increased TUNEL with p≤0.05; − − = significantly decreased TUNEL with p≤0.01; − = significantly decreased TUNEL with p≤0.01, NC = no significant change in TUNEL).

### RNAi Screen Identifies Novel Genes that Affect Cell Survival and Cell Death

**Figure 2:**
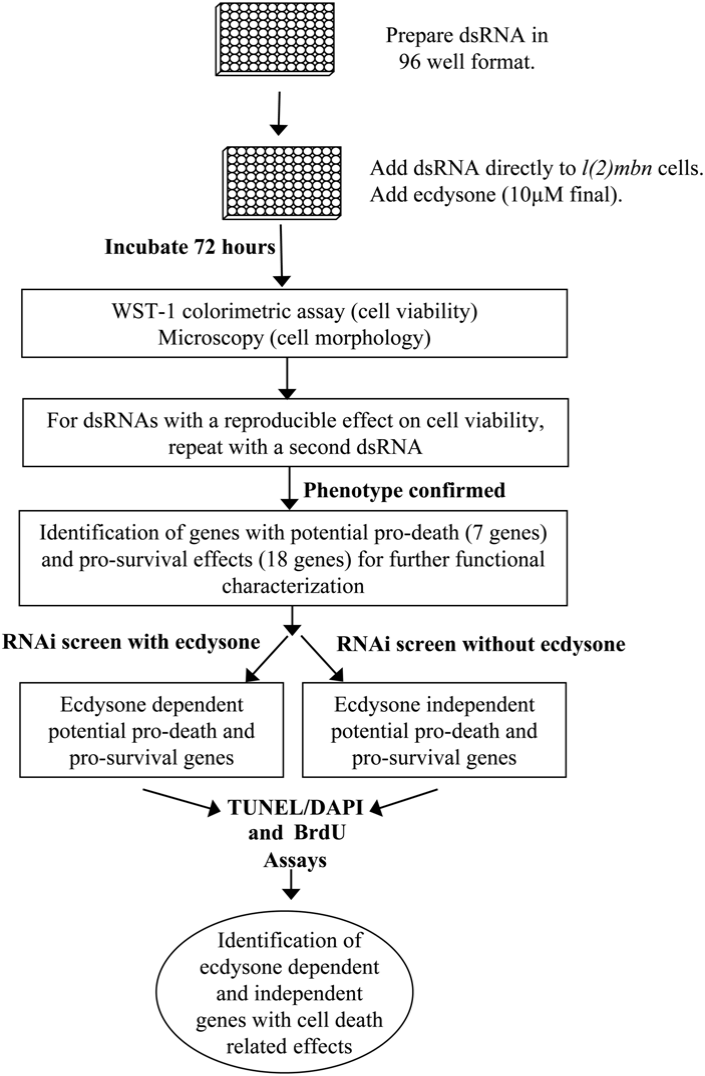
Overview of RNAi screen in *l(2)mbn* cells. A set of 460 genes which included known transcription factors, cell death and autophagy genes, as well as genes associated transcriptionally with ecdysone-induced cell death of the *Drosophila* salivary gland were chosen for this functional study. In our initial screen, cells were treated with ecdysone and dsRNA corresponding to each of these genes. A viability assay (WST-1) was used to identify genes with potential pro-death or pro-survival functions, and microscopy was used to visualize cell morphology. For the reproducible positive hits, a second dsRNA was used to confirm the viability phenotype. The screen identified 7 potential pro-death and 18 pro-survival genes. The RNAi/WST-1 screen was repeated with and without ecdysone to determine ecdysone dependency for the 25 identified genes. TUNEL/DAPI and BrdU assays were performed to identify genes with cell death and/or cell proliferation related effects.

To identify additional genes that function in ecdysone-mediated cell death or cell survival, we conducted an RNAi screen (Figure 2). Based on genome-wide transcript and protein expression studies conducted previously in *Drosophila* larval salivary glands [23],[24],[40], there are a large number of genes and proteins that could affect ecdysone-mediated PCD but have not been tested functionally. Here, we conducted a systematic study of 460 of these genes which included all of the annotated genes from our previous study [24] that showed a significant (p≤0.05 and 5 fold difference) increase or decrease in expression levels in salivary glands immediately prior to PCD. The WST-1 assay was used as a primary screen to assess effects of the 460 dsRNAs on cell viability (Table S1). Using this assay, we identified five genes reported already to have a pro-survival role based on a previous RNAi screen [47],[49],[57]; (Table 1 and Table S1). In addition, we identified and validated another 20 genes with corresponding dsRNAs that significantly increased or decreased cell viability (Table 1). All of these 20 genes were validated with completely non-overlapping dsRNAs. In total, our final gene set for further analyses consisted of 18 genes with corresponding dsRNAs that resulted in reduced viability (hereafter referred to as pro-survival genes) and 7 genes with corresponding dsRNAs that resulted in increased viability (hereafter referred to as candidate pro-death genes).

### Identified Pro-Survival Genes Act in an Ecdysone-Dependent or Ecdysone-Independent Manner

**Figure 3:**
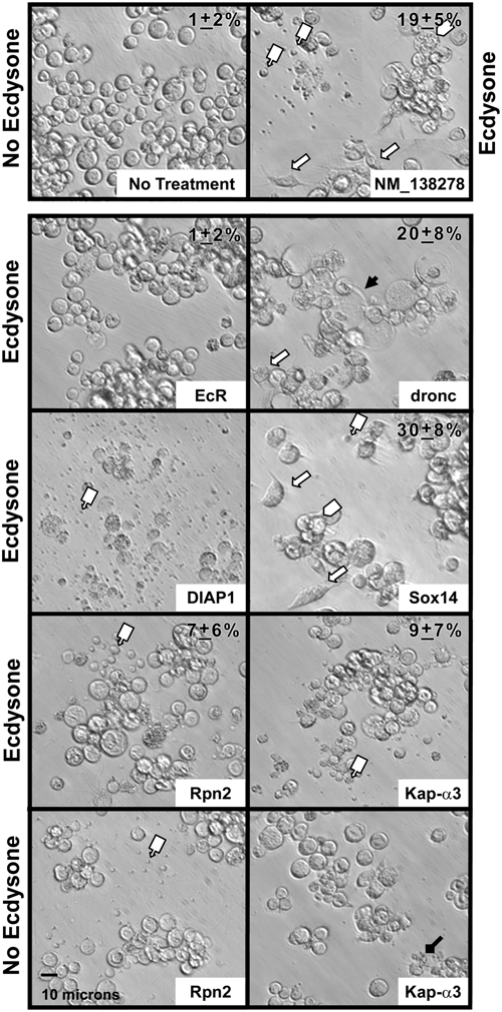
Cellular morphology of *l(2)mbn* cells after dsRNA treatment. Cellular phenotypes were visualized 3 days after dsRNA treatment in the presence or absence of ecdysone. The *l(2)mbn* cells with no ecydsone and no dsRNA (No treatment) were round and uniform in size and shape. *l(2)mbn* cells treated with ecdysone (not shown) or ecdysone+human dsRNA NM_138278-negative control changed in shape, from round to spindle forms with extensions (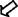) (19+/−5%). Large cells with phagocytosed material (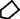) and apoptotic bodies (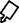) were also observed. RNAi of *EcR*, *dronc* and *Sox14* each increased viability of the ecdysone treated cells, but their resulting morphologies were distinct. The % of observed spindle shaped cells (top right corner) was quantitated for dsRNAs corresponding to the genes indicated. RNAi of *EcR* inhibited spindle shape formation (1+/−2% of the cells were spindle shaped), cells remained rounded, and no apoptotic bodies were found. RNAi of *dronc* inhibited apoptotic body formation, but cells became spindle shaped (20+/−8%). Also, signs of necrosis such as inflated and seemingly empty cells and cell fragments were observed (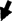). RNAi of *Sox14* showed few apoptotic bodies and 30+/−8% of the cells were spindle-shaped. RNAi of *diap1* resulted in formation of numerous apoptotic bodies within 24 hr and no spindle shaped cells were found. RNAi of *Rpn2* showed numerous apoptotic bodies in the presence and absence of ecdysone. while RNAi of *Kap-a3* showed a dramatic increase in apoptotic bodies only in the presence of ecdysone (see Table 2 for quantitation of TUNEL positive cells).

To determine which genes are regulated by the ecdysone signaling pathway, we investigated whether the decreased cell viability phenotype caused by RNAi knock-down of the 18 pro-survival gene products was ecdysone dependent. We treated the cells with dsRNA and assessed cell viability with and without ecdysone treatment. This analysis resulted in the identification of five ecdysone dependent pro-survival genes (*CG33087*, *CG13784*, *CG32016*, *Ras85D*, *Cp1*; Table 1). dsRNAs corresponding to these five genes reduced cell viability only in the presence of ecdysone and did not affect viability of *l(2)mbn* cells in the absence of ecdysone. Of the five genes identified, three (*CG33087*, *CG13784*, *CG32016*) were uncharacterized previously. Of these three genes, two (*CG13784*, *CG32016*) do not have any recognizable protein domain or predicted gene function (FlyBase) [58]. We confirmed the ecdysone dependent pro-survival effect of two (*CG32016*, *Cp1*) of the 5 identified genes in another *Drosophila* cell line, S2; the other three ecdysone dependent genes identified in *l(2)mbn* cells did not significantly affect S2 cell viability in the presence of ecdysone (Table S2). dsRNA corresponding to 13 other genes (Table 1) reduced viability of *l(2)mbn* cells following ecdysone treatment. However, a decreased viability phenotype, as assessed by WST-1, was also observed for these 13 dsRNAs in *l(2)mbn* cells in the absence of ecdysone. Nine of the 13 dsRNAs showed similar viability effects in S2 cells in the presence or absence of ecdysone (Table S2). We initially categorized the 13 genes as ecdysone independent pro-survival genes. Among this group of genes, dsRNA corresponding to *Kap-α3* resulted in different phenotypes, as assessed initially by cell morphology (Figure 3), in the absence and presence of ecdysone. In the absence of ecdysone, *Kap-α3* dsRNA did not result in detectable apoptotic bodies (up to 72 hrs), but in the presence of ecdysone and as early as 48 hrs following treatment, the same dsRNA resulted in a dramatic increase in apoptotic bodies compared to controls (Figure 3; Table 2). This result indicates that while the overall survival effect of this gene product may be ecdysone independent, its mechanism of action differs depending on the presence or absence of ecdysone.

### TUNEL and BrdU assays distinguish pro-survival genes associated with cell death inhibition

**Figure 4:**
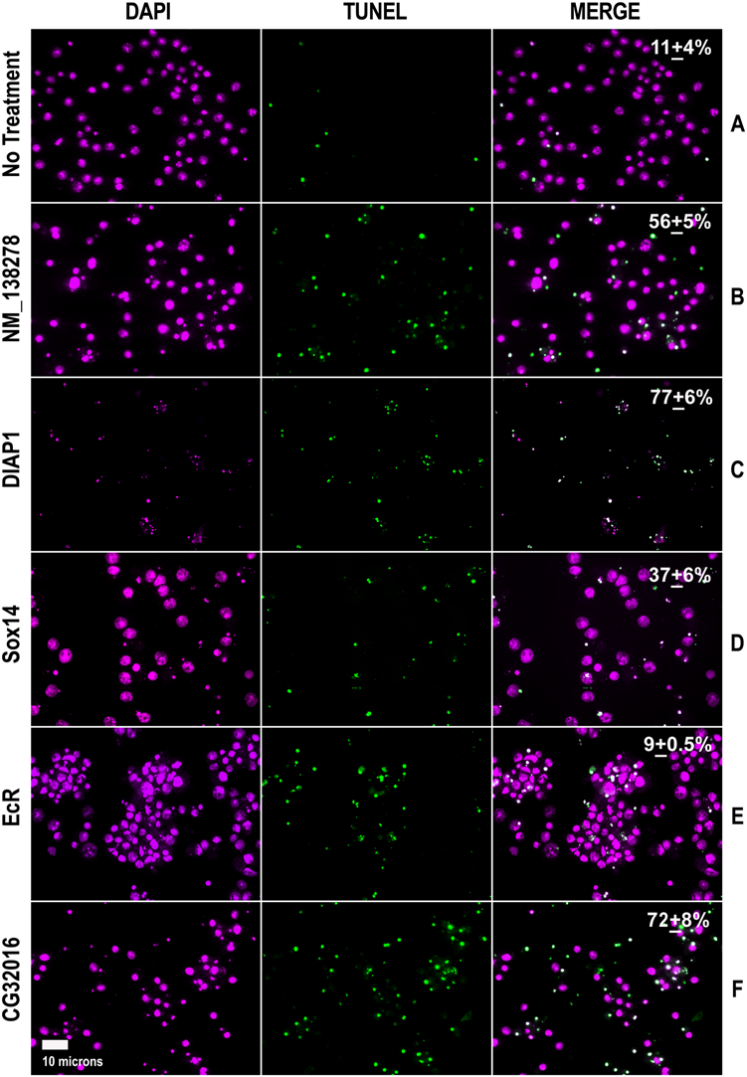
TUNEL assay identifies genes with cell death-related effects. DAPI staining (magenta) and TUNEL assays (green) were performed 3 days following addition of dsRNA and ecdysone to *l(2)mbn* cells. The cells with no ecdysone and no dsRNA (No treatment) showed a background level of 11+/−4% TUNEL positivity (Panel A). Ecdysone (not shown) and ecdysone plus the human dsRNA NM_138278-negative control resulted in an increase in TUNEL positive cells (56+/−5%; Panel B). dsRNA corresponding to *diap1* (control gene) increased TUNEL positive cells without (not shown) or with ecdysone treatment (77+/−6%; Panel C). Ecdysone plus RNAi of *Sox14* (Panel D) or *EcR* (Panel E) resulted in decreased TUNEL positive cells (37+/−6% and 9+/−0.5%, respectively), whereas ecdysone plus RNAi of a novel gene *CG32016* (Panel F) increased TUNEL positive cells (72+/−8%) compared to the negative control.

To determine whether the decreased viability of cells treated with ecdysone and dsRNA corresponding to the pro-survival genes is due at least in part to increased cell death, we performed the TUNEL/DAPI assay for representative genes from this category. We treated cells with dsRNA of two ecdysone dependent (*CG32016* and *Ras85D*) and six ecdysone independent (*Kap-α3*, *Pros26.4*, *Smr*, *Sin3A*, *S6K*, *and Tor*) genes in the presence and absence of ecdysone and quantified the percent TUNEL positive cells. RNAi of six genes (*CG32016*, *Ras85D*, *Kap-α3*, *Pros26.4*, *Smr*,*and S6K*) increased significantly the percentage of TUNEL positive cells (p≤0.05) in the presence of ecdysone (Groups A and B, Table 2, Figure 4), indicating a potential death inhibitory pro-survival role. RNAi of *Sin3A* and *Tor* did not significantly (p>0.05) increase the percentage of TUNEL positive cells in the presence of ecdysone, indicating that their pro-survival effects in this context are likely not due to an inhibition of cell death (Group C, Table 2). However, RNAi of these same two genes did result in an increase in percent TUNEL positive cells in the absence of ecdysone compared to the controls (Table 2). In contrast, our TUNEL/DAPI assay indicated that knock-down of *Kap-α3* and *Smr* by RNAi increased TUNEL positive cells only in the presence of ecdysone (Group A, Table 2). This result is in agreement with the previously observed increase in apoptotic bodies found only in the presence of ecdysone (Figure 3). The reduced viability caused by RNAi of *Kap-α3*, and *Smr* in the absence of ecdysone appears not to be death-related and may instead be due to inhibition of cell proliferation. To test this possibility, we conducted a BrdU incorporation assay which indicated reduced proliferation in *Kap-α3*-RNAi but not in *Smr*-RNAi treated cells compared to control-RNAi treated cells (p<0.05; Figure S2). RNAi of *Pros26.4* and *S6K* resulted in an increase in TUNEL positive cells (p≤0.05) both in the absence and presence of ecdysone, distinguishing them as ecdysone independent and potential negative regulators of cell death. The TUNEL/DAPI assay also confirmed the ecdysone dependent and potential death inhibitory survival role of *CG32016* and *Ras85D* (Group A, Table 2). Consistent with WST-1 findings, the BrdU incorporation assay for these two dsRNAs indicated no significant change in proliferation (p = 0.5 and 0.4, respectively; Figure S2) in the absence of ecdysone. In summary, based on WST-1, TUNEL and BrdU assays, we conclude that *CG32016*, *Ras85D*, *Kap-α3*, *and Smr*, are ecdysone dependent pro-survival genes that result in decreased cell death, and *Pros26.4*, *S6K*, *Tor*, and *Sin3A* are ecdysone independent pro-survival genes that result in decreased cell death. The observed effects on cell death following RNAi of these genes may be directly or indirectly related to gene function.

### TUNEL Assay Validates Genes with a Pro-Death Function in Ecdysone-Mediated *l(2)mbn* Cell Death

Our RNAi study identified seven candidate pro-death genes, comprised of two 40S ribosomal genes (*RpS5* and *RpS6*), three 60S ribosomal genes (*RpL13A*, *RpL37* and *RpLP1*), one transcription factor Sox box protein (*Sox14*) and one sorting nexin-like gene (*SH3PX1*). To determine whether their potential pro-death effects are ecdysone dependent, we performed RNAi assays with and without ecdysone. Consistent with observations by others [49], dsRNAs corresponding to the ribosomal genes had the opposite effect in the absence of ecdysone, resulting in a significant reduction in cell viability (Table 1, column 3, bold and italicized) when compared to control cells. In agreement with the cell viability assay, the BrdU assay showed reduced proliferation in the ribosomal gene-RNAi treated cells in the absence of ecdysone (p<0.05; Figure S2). To confirm the putative pro-death role of the ribosomal genes observed in the presence of ecdysone in *l(2)mbn* cells, we employed the TUNEL/DAPI assay as described above. Knock-down of all ribosomal genes tested, with the exception of *RpS6*, resulted in a decrease in the percent TUNEL positive cells (Table 2) following ecdysone treatment, indicating that *RpS5*, *RpL13A*, *RpL37* and *RpLP1* have a pro-death related function in *l(2)mbn* ecdysone-mediated death. The TUNEL/DAPI assay also indicated that the transcription factor *Sox14*, and the sorting nexin-like gene *SH3PX1* act as pro-death genes (Table 2). Therefore, our RNAi study which employed both cell viability (WST-1) and cell death TUNEL/DAPI assays identified six new genes (*RpS5*, *RpL13A*, *RpL37* and *RpLP1*, *SH3PX1*, *Sox14*) required for ecdysone-mediated cell death in *l(2)mbn* cells.

### Transcription Factor Sox14 Is Induced by Ecdysone and Its Overexpression Is Sufficient to Induce Apoptosis *In Vitro*

**Figure 5:**
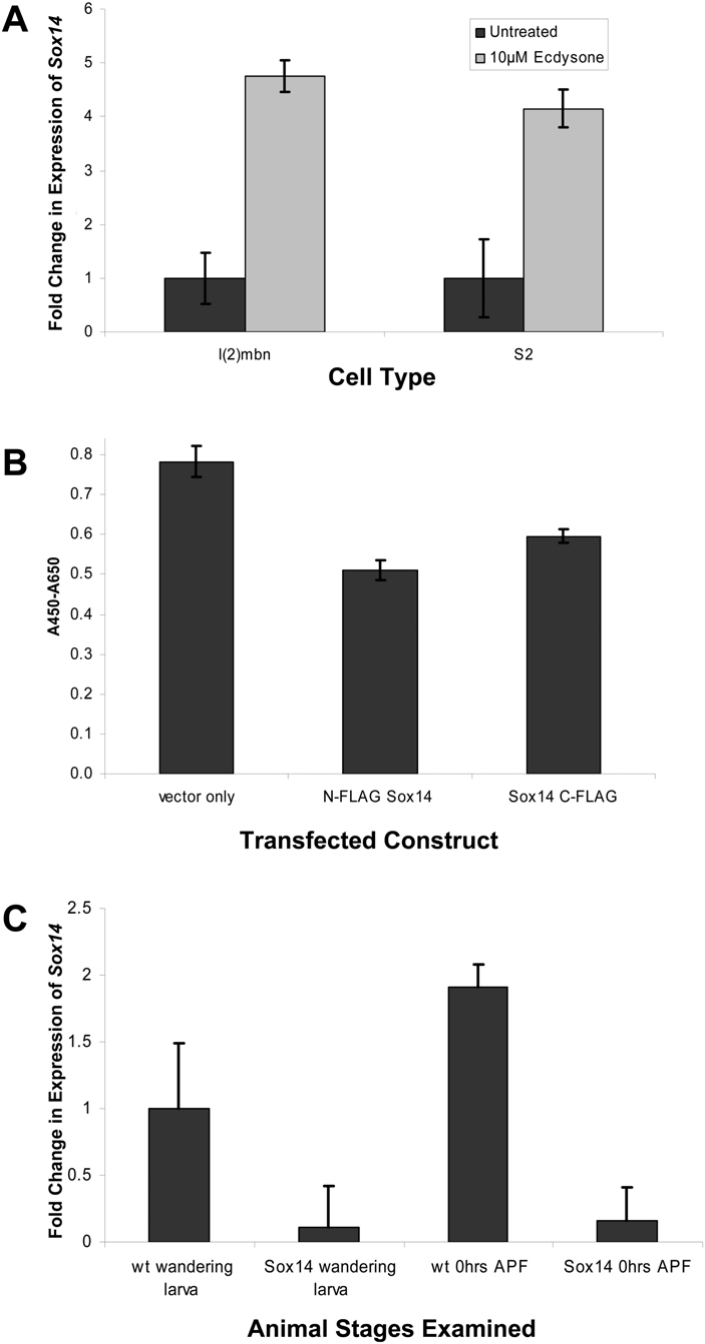
Expression analyses of *Sox14* in *Drosophila* cell lines and *Sox14-RNAi* animals. (A) QRT-PCR expression profiling of *l(2)mbn* and S2 cells treated with 10 µM ecdysone for 72 hrs showed a 5 fold and 4 fold increase, respectively, in *Sox14* transcripts compared to untreated control cells. Expression of *Sox14* was normalized to the housekeeping gene *rp49*. (B) *l(2)mbn* cells transfected with either N- or C-terminal FLAG tagged Sox14 constructs showed decreased cell viability after 48 hrs compared to cells transfected with the control vector only (p≤0.001). (C) *Sox14* expression in wild-type and *Sox14-RNAi* whole animals. In control wild type (wt; strain *w^1118^*) whole animals, increased transcript levels of *Sox14* were observed at 0 hrs APF compared to wandering larvae. In *Tub-Sox14-RNAi (Tubulin-GAL4/Sox14-RNAi)* animals, *Sox14* transcripts were dramatically reduced at both stages compared to the control (89+/−2% and 91+/−2% reduction in transcript levels at the wandering larva and 0 hrs APF stages, respectively). Error bars in (A)–(C) represent the SD of triplicate samples.

To determine whether *Sox14* expression is induced by ecdysone, we treated both *l(2)mbn* and S2 cells with ecdysone and determined the expression levels of *Sox 14* by QRT-PCR. As shown in Figure 5A, ecdysone treatment resulted in a 5 fold and 4 fold increase in expression of *Sox14* in *l(2)mbn* and S2 cells, respectively.

To determine whether Sox14 is sufficient to decrease cell viability, we overexpressed C and N-terminal FLAG tagged Sox14 protein in *l(2)mbn* cells and measured cell viability using the WST-1 assay. Overexpression of Sox14 reduced cell viability, detectable 48 hrs following transfection (Figure 5B). By approximately 96 hrs after transfection, apoptotic bodies were evident in Sox14 overexpressing cell cultures but not in control cells transfected with empty vector (data not shown). These results indicate that Sox14 expression is sufficient to induce apoptosis in *l(2)mbn* cells.

### *Sox14-RNAi* Animals Have Defects in Larval Midgut and Salivary Gland Destruction

**Figure 6:**
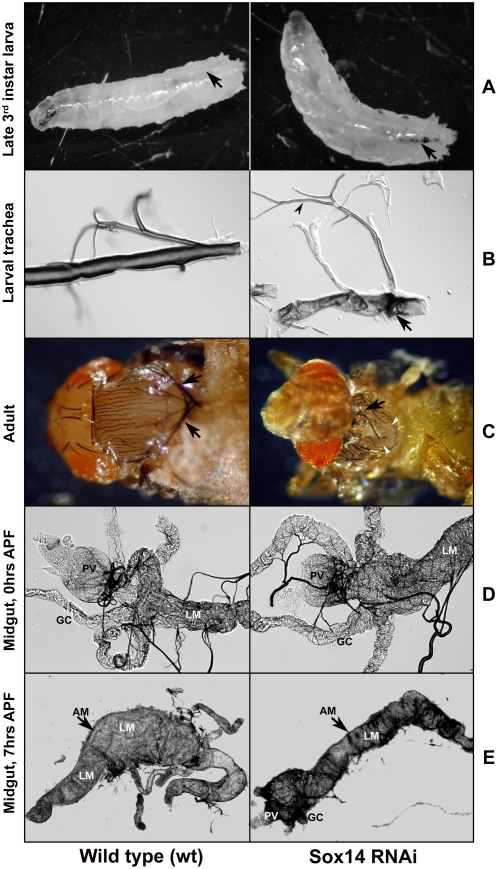
*Sox14-RNAi* animals have multiple developmental abnormalities including defects in larval midgut destruction. Control wt late 3rd instar larva show normal trachea (left, arrow) but *Sox14-RNAi* late 3rd instar larva displayed tracheal defects detectable as blackened regions (right, arrow). (B) Dorsal tracheal trunks of *Sox14-RNAi* animals (right) showed severely distorted taenidial folds, collapse of the tracheal cuticle and blackening of the cuticle (arrow). Branching of the trachea in *Sox14-RNAi* animals appears to occur normally (arrowhead) as in control wt animals (left). (C) *Sox14-RNAi* pharate adults dissected from the pupal case showed defects in eyes, notum, and bristles (right, arrow). A control wt pharate adult with normal eyes, notum and bristles (arrows) is shown on the left for comparison. (D) In the 0 hr APF larval midgut (LM) from both control wt (left) and *Sox14-RNAi* animals (right), the proventriculus (PV) and gastric caecae (GC) are evident. (E) In wt animals, the midgut condensed by 4 hrs APF (not shown) and the gastric caecae were not detectable by 7 hrs APF. Condensed wild type larval midgut (LM) was found within the adult midgut by 7–12 hrs APF (left). *Sox14-RNAi* animals showed some condensation of the midgut but the proventriculus (PV) and remnants of gastric caecae (GC) can still be observed even after 7–12 hrs APF (right). In both wt and *Sox14-RNAi* animals, a layer of adult midgut (AM, arrow) can be observed after 4 hrs APF.

To examine the function of Sox14 *in vivo*, we used a *Tubulin-GAL4* driver (*Tub*-*GAL4*) to ubiquitously express *Sox14* dsRNA (*Tub-GAL4/Sox14-RNAi*; referred to as *Tub-Sox14-RNAi*). QRT-PCR analysis using RNA from *Tub-Sox14-RNAi* wandering larvae and 0 hrs APF pupae showed a reduction in *Sox14* transcripts of 89+/−2% and 91+/−2%, respectively, compared to wild-type control animals (Figure 5C). The *Tub-Sox14-RNAi* animals demonstrated lethality at 3rd instar larval, pupal or pharate adult stages. During pupation, the *Tub-Sox14-RNAi* animals displayed three distinct lethal phases: 14% died during early prepupal development (i.e. prior to head eversion), 74% died during early to mid pupal development (showed head eversion and/or leg elongation) and 12% died during the pharate adult stage (n = 104 pupae). In *Tub-Sox14-RNAi* larvae, defects in the trachea were observed (Figure 6A). The branching of the tracheal system appeared normal but the dorsal tracheal trunks showed severely distorted taenidial folds, collapse of the tracheal cuticle and blackening of the cuticle (Figure 6B). *Tub-Sox14-RNAi* animals did not eclose, but we dissected out pharate animals and found obvious alterations in the notum (malformed; split) and bristles (missing and mis-oriented) (Figure 6C). The Sox14-related cellular alterations giving rise to these defects remain to be determined.

To initiate investigations of *Sox14* in programmed cell death, we first examined the larval midgut (Figure 6D and E); this tissue was examined since most (86%) *Tub*-*Sox14-RNAi* pupae persist past the normal stage of larval midgut cell death. By 4 hrs APF, the proventriculus is significantly reduced in size and the gastric caeca are no longer detectable in wild-type animals. Head eversion occurs at approximately 10–12 hrs APF, at which time point the larval midgut is entirely destroyed, compressed and surrounded by the adult midgut [9]. As expected, in control animals (*Tub-GAL4/+*; designated wild-type or wt) we observed midgut condensation by 4 hrs APF and the gastric caecae were not detectable after 7 hrs APF (n = 10) (Figure 6E). The wild type larval midgut appeared degraded and the remnants were found within the adult midgut by 12 hrs APF (n = 5). Similar to *BR-C* mutants [10], the *Tub*-*Sox14-RNAi* pupae showed some condensation of midguts, but a remaining proventriculus and remnants of gastric caecae were still observed even after 7–12 hrs APF (n = 12) (Figure 6E). A remaining proventriculus and gastric caecae remnants were observed even in animals that had clearly undergone head eversion (ie. 10–12 hrs APF). These observations indicate that reduced *Sox14* expression results in partially defective larval midgut cell death and thus Sox14 is normally required for complete destruction of the larval midgut.

**Figure 7:**
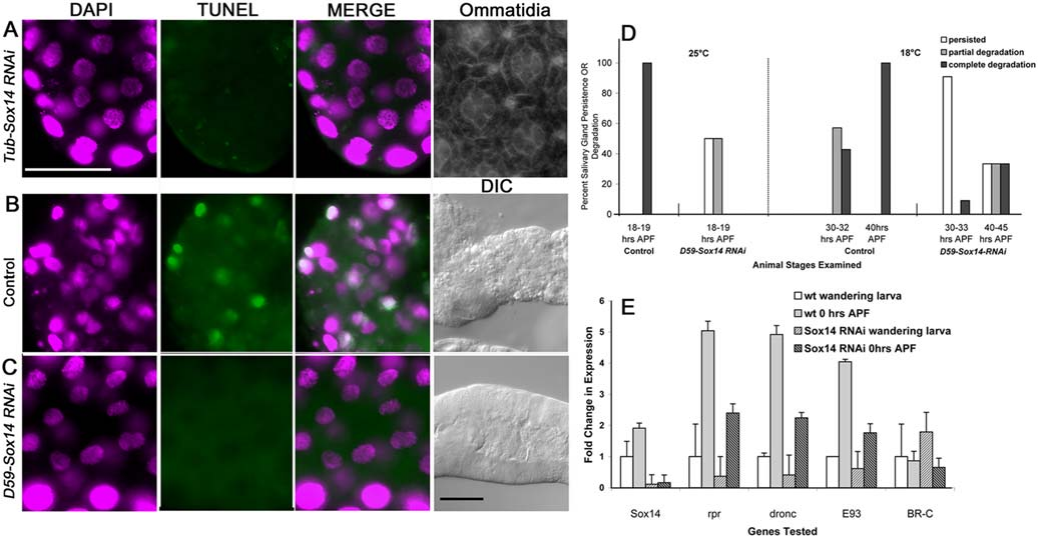
*Sox14-RNAi* results in a delay or inhibition of salivary gland cell death. A. In *Tub-Sox14-RNAi* pupae, intact salivary glands persisted past the stage of wild-type salivary gland cell death (15–18 hrs APF at 25 C). Shown is a segment of an intact salivary gland from a *Tub-Sox-14-RNAi* pupa that is devoid of TUNEL positive nuclear staining (DAPI = magenta; TUNEL = green). Phalloidin staining of retinas dissected from the same animal in (B) was used as an independent morphological marker to help determine developmental age. The ommatidial arrangement shown (far right) indicates that the animal developed to at least 30 hrs APF (25 C). B. In control pupae (*D59-GAL4/D59-GAL4*; *MKRS/TM6B*), extensive nuclear TUNEL staining (DAPI = magenta; TUNEL = green) and degradation (DIC) was evident at stages equivalent to 16–17 hrs APF at 25 C (or 30–32 hrs APF at 18 C). C. In *D59-Sox14-RNAi* pupae, salivary gland cell death was delayed. Shown is a salivary gland dissected at the same stage as the animal in (B). There is no evidence of TUNEL positive (green) nuclei (DAPI = magenta) and no indications of degradation (DIC). Scale bars for fluorescent and DIC images in A–C = 100 microns. D. Quantitation of salivary gland degradation and persistence in control and *D59-Sox14-RNAi* pupae raised at 25 C and 18 C. In control animals at 25 C, no salivary glands were detected by 18–19 hrs APF. In *D59-Sox14-RNAi* animals dissected at 18–19 hrs APF, completely intact salivary glands (persisting salivary glands) were found in 50% of the animals, and some degradation was observed (partial degradation) in the remaining 50%. By 21 hrs APF (25 C), salivary glands were not detected in this strain. In control animals at 18 C, 57% showed partial salivary gland degradation and 43% showed complete degradation by 30–32 hrs APF; salivary glands were not detected by 40 hrs APF. In *D59-Sox14-RNAi* pupae at 18 C, 91% of the animals had persisting salivary glands at 30–33 hrs APF. At 40–45 hrs APF, one-third of the animals had intact salivary glands, one-third had partially degraded salivary glands, and the remaining third had no detectable salivary glands. Salivary glands were examined from at least 10 animals at each time point and at each temperature. E. QRT-PCR analysis of gene expression in control wild-type (*w^1118^*) and *Tub-Sox14-RNAi* (*Tubulin-GAL4/ Sox14-RNAi*) animals. In *Tub-Sox14-RNAi* animals, *Sox14* transcripts were reduced at both stages compared to the control. In control animals, apoptosis genes *rpr* and *dronc* and the ecdysone regulated transcription factor gene *E93* showed a 4 to 5 fold increase in expression at 0 hrs APF compared to wandering larvae. However, the transcript levels corresponding to these genes showed a reduction at both stages in the *Tub-Sox14-RNAi* strain compared to the control strain. The ecdysone regulated transcription factor *BR-C* did not show expression level differences between wild-type and *Tub-Sox-14-RNAi* animals at the stages examined. Error bars represent the SD of triplicate samples.

To examine the role of Sox14 in salivary gland cell death, we first examined salivary glands from head-everted *Tub-Sox14-RNAi* pupae (n = 23; equivalent to >30 hrs APF at 25°C based on incubation time). At this timepoint, all 25 animals still had intact salivary glands. However, since *Tub-Sox14-RNAi* animals arrest at various developmental ages following head eversion, we used retinal pattern formation [59] as an independent morphological marker to aid in the developmental staging. Retinae were dissected and stained with phalloidin to visualize ommatidial patterning [60]. All 23 animals had fully everted eye discs consistent with development to at least 12 hrs APF (at 25°C), and 8 animals had retinas with ommatidial patterning indicative of development to at least 22 hrs APF at 25°C [59]. Of these 8 animals, ommatidal patterning indicated that 5 developed to at least 30 hrs APF at 25°C (e.g. Figure 7A). In rare instances (n = 6 out of more than 100 pharate adults dissected), we were able to dissect intact salivary glands from *Tub-Sox14-RNAi* pharate adults with darkened wings and red eyes indicative of development to approximately 100 hrs APF (25°C ) [59]. To further analyze Sox14 function in salivary gland cell death, we employed a salivary gland GAL4 driver (*D59-GAL4*) to express *Sox14* dsRNA. A single copy of the driver did not result in a phenotype, but two copies of the driver (*D59-GAL4/D59-GAL4*; *Sox14-RNAi/TM6B*) resulted in a delay in salivary gland cell death compared to control animals (*D59-GAL4/D59-GAL4*; *MKRS/TM6B*) (Figure 7B–D). In the control animals, TUNEL positive nuclei were prevalent in salivary glands equivalent to 16–17 hrs APF at 25°C (ie. 30–32 hrs APF at 18°C ) but were not observed in salivary glands from *D59-Sox14-RNAi* or *Tub-Sox14-RNAi* animals at a comparable or later stage, respectively (Figure 7A–C). Together, these results indicate that reduced levels of Sox14 expression result in either a delay or inhibition of salivary gland cell death, and thus Sox14 functions as a positive regulator of salivary gland cell death.

To help place Sox14 within the context of the known signaling pathways required for ecdysone-mediated cell death, we examined gene expression in *Tub-Sox14-RNAi* animals. Transcript levels of two apoptosis effectors, *rpr* and *dronc*, and two ecdysone regulated transcription factors, *E93* and *BR-C*, were examined in *Tub-Sox14-RNAi* wandering larvae and 0 hr APF pupae and compared to controls. While *BR-C* showed no changes in expression levels between control and *Tub-Sox14-RNAi* animals at both stages examined, *rpr*, *dronc* and *E93* transcripts were reduced in *Tub-Sox14-RNAi* 0 hr APF pupae compared to controls (Figure 7E). These results support a pro-death role for Sox14, and indicate that Sox14 acts upstream of rpr, dronc and E93 and either downstream or in parallel to BR-C. Future studies are required to determine whether Sox14 directly regulates the transcription of any of these genes, and whether the hierarchical position of Sox14 is conserved in various developmental stages and tissues.

## Discussion

We performed an RNAi screen as a means of gaining new molecular insights into ecdysone induced cell death and cell survival signaling pathways. We enriched for the identification of ecdysone-dependent genes by targeting genes that were differentially expressed in *Drosophila* larval salivary glands immediately prior to ecdysone-induced cell death 23,24. In total, we verified functionally the pro-death effects of six genes and the pro-survival effects of 18 genes, and further characterized their functions on the basis of ecdysone dependency and cell death effects. More detailed examination of one gene, *Sox14*, showed that it was induced by ecydsone and its expression was sufficient to induce apoptosis *in vitro*. Studies *in vivo* revealed a role for Sox14 in larval midgut and salivary gland cell death.

Potential off-target effects can be a significant issue in any RNAi screen especially when long dsRNAs are used [61],[62]. Although *Drosophila* does not have interferon responses as observed in mammals, short dsRNAs (≥19 nt) produced by Dicer processing that are perfect matches to non-target specific transcripts are the likely source of off-target effects [61]–[64]. To help eliminate potential false positives due to off-target effects or experimental noise, we designed a second dsRNA, free of predicted off-target effects and completely non-overlapping with the first dsRNA [65] (in all but one case – see Materials and Methods). For a gene to be considered further, both of its dsRNAs had to produce an effect in the same direction with a p-value of ≤0.05. While we may have eliminated some false negatives due to insufficient RNAi knockdown by this screening strategy, these stringent criteria enabled us to produce a highly reliable final list of candidate genes for further study. Many, but not all, of the dsRNAs corresponding to these candidate genes had similar effects on viability in *l(2mbn)* and S2 cell lines (Table S2). The observed differences may be attributable to the different genotypes of these cell lines. Since both *l(2)mbn* and S2 cell lines are polyclonal, we also cannot rule out the possibility of an inhomogenous response to the dsRNAs tested. This could affect the overall detectable response to RNAi treatment and thus is another possible reason why results could differ in these or alternate cell lines.

Recently, a role for *Drosophila* autophagy genes *atg1*, *atg2*, *atg3*, *atg6*, *atg7*, *atg8a*, and *atg12* in salivary gland degradation has been demonstrated [66]. Our study did not find a death related role for autophagy genes in *l(2)mbn* cells in the presence of ecdysone. It is possible that these genes do not have an essential death or survival related role under the conditions we tested. Since our screen was optimized to detect effects of genes that are dependent on ecdysone-regulated transcription, we cannot rule out the possibility that additional genes impacting ecdysone-mediated PCD may be detected under different experimental conditions. However, in the absence of ecdysone, knock down of several Atg genes (*atg2*, *atg3*, *atg5*, *atg6*, *atg7*, *atg8a*, *atg8b*) resulted in decreased cell viability (Figure S1) indicating a potential pro-survival role for these genes.

Our screen was validated by identification of known genes and biochemical complexes with previously established cell survival or cell death phenotypes. For example, *Ras85D* promotes cell survival in *Drosophila* by down-regulating *hid* expression and activity [67],[68] *in vivo*. Consistent with these findings, we discovered that decreased *Ras85D* transcripts resulted in reduced cell survival in an ecdysone dependent manner, while knockdown of *hid* resulted in a phenotype of increased cell survival. These results suggest that Ras pathway mediated inhibition of Hid activity may exist in the ecdysone signaling pathway. We also identified Smr, a co-repressor, and dSin3A, a transcriptional regulator, that associate with each other to mediate the transcriptional silencing of the EcR∶USP complex. Addition of ecdysone completely dissociates Smr from the EcR∶USP heterodimer complex and activates EcR∶USP mediated transcription. Elimination of repression by Smr/Sin3A on EcR∶USP activity resulted in lethality *in vivo* [69]. Based on these observations, we predicted that reduced expression of either Smr or Sin3A or both by RNAi in our system would release, as with ecdysone, the repression caused by these gene products on the EcR∶USP complex, resulting in increased EcR∶USP activation and subsequent increased cell death. As we expected, our cell viability/TUNEL assays in *l(2)mbn* cells indicated clearly that knock-down of *Smr* transcripts resulted in increased cell death in an ecdysone dependent manner (Table 2). The identification of such known ecdysone signaling complexes demonstrates that our assay is a viable method for functional verification and initial characterization of genes involved in ecdysone-mediated death/survival pathways

The predicted or known function of several pro-survival genes identified in our screen (*Pros26.4*, *Rpn2*, *Tbp-1 and Cp1*) was associated with protein degradation processes. Under stress conditions, down regulation of gene products associated with protein degradation processes could impair energy production and, therefore, reduce the survival of the cell/organism. The 26S proteasome complex, a major site of protein degradation, is made up of two multi-subunit sub complexes, namely the 20S Proteasome and PA700 (19S complex). The identified pro-survival genes, *Pros26.4*, *Rpn2*, and *Tbp-1* all belong to the PA700 subunit of the 26S proteasome complex. Proteasome function is required for cell proliferation [70] and silencing the expression of gene products belonging to the PA700 complex by RNAi reduced cell proliferation and induced apoptosis in S2 cells [49],[71]. Consistent with these previous findings, our results indicated that *Pros26.4*, *Rpn2*, and *Tbp-1* knockdown led to reduced viability of *l(2)mbn* and S2 cells both in the presence and absence of ecdysone. In our RNAi screen, the pro-survival genes that were associated previously with protein degradation (as above) or protein transport (Kap-α3) were significantly up-regulated prior to larval salivary gland histolysis [24]. During PCD, anabolic processes are reduced and, therefore, a replenishable source of carbohydrates is unavailable for energy production. Thus, it is possible that ecdysone may activate protein degradation processes in salivary glands to produce energy to complete the death process.

Our RNAi screen identified five previously uncharacterized genes (*CG13784*, *CG15239*, *CG32016*, *CG33087*, *and CG7466*) as pro-survival genes. Among these, *CG13784*, *CG32016* and *CG33087* were ecdysone dependent for their pro-survival role. Further studies are required to determine whether these three genes affect survival in response to other agents that induce cell death; our preliminary data (not shown) indicates that they do not have any effects on staurosporine induced cell death. The products of *CG33087* (calcium ion binding; ATPase activity; low-density lipoprotein receptor activity) and *CG7466* (receptor binding; cell-cell adhesion) have predicted functions based on protein domains but *CG13784*, *CG15239*, and *CG32016* have no illuminating sequence characteristics. We further characterized *CG32016* in *l(2)mbn* cells by the TUNEL assay in both the presence and absence of ecdysone. Knock-down of *CG32016* resulted in increased TUNEL positive cells only in the presence of ecdysone, indicating a potential cell death-related, ecdysone-dependent pro-survival role. We are the first to associate a function with these previously uncharacterized gene products (CG13784, CG15239, CG32016, CG33087, and CG7466); additional studies will be required to elucidate their specific positions and functions in response to ecdysone.

Of the 25 genes that were identified in our screen, seven genes (Table 1) were identified as potential pro-death genes. Of these seven genes, five were ribosomal genes. In *Drosophila*, 38 small (40S) and 49 large (60S) ribosomal proteins have been identified [72]; the small ribosomal subunits belong to the eukaryotic pre-initiation complex and the large ribosomal subunits are usually involved in translation. We tested in our RNAi screen the five ribosomal genes that were differentially expressed in the *Drosophila* larval salivary glands immediately prior to PCD [24]. RNAi of both small ribosomal genes (*RpS5*, *RpS6*) and large ribosomal genes (*RpL13A*, *RpL37 and RpLP1*) resulted in increased cell viability of ecdysone treated *l(2)mbn* cells, indicating that these genes may have a pro-death role in the presence of ecdysone. Further, with staurosporine treatment (data not shown), RNAi of these ribosomal genes resulted in reduced cell viability, indicating that ecdysone is indeed required for the increased viability effect of these dsRNAs *l(2)mbn* cells. Ecdysone treatment induces transcription of pro-death genes such as *BR-C*, *dronc*, *rpr* and *hid*, and ribosomal gene products are required for their translation. Thus, knocking down ribosomal gene products by RNAi may affect efficient translation of pro-death genes leading to the observed phenotype of increased viability. However, in S2 cells, knock-down of these ribosomal genes in the presence of ecdysone did not increase cell viability but rather significantly decreased viability (Table S2); further studies are required to understand these cell line dependent effects. In the absence of ecdysone, RNAi of these same ribosomal genes resulted in reduced viability in both *l(2)mbn* and S2 cells, supporting a pro-survival role under these conditions. This pro-survival effect is similar to that reported in S2 and Kc cells by others [47],[49]. A pro-survival function of ribosomal proteins in the absence of ecdysone is in agreement with the key role they play in protein-synthesis and, therefore, in cell growth and cell proliferation.

Our screen identified two additional gene products required for ecdysone-mediated cell death: i) dSH3PX1, involved in intracellular protein transport and resembling a sorting nexin with an NH2-terminal SH3 domain and a central phox homology (PX) domain [73],[74], and ii) Sox box protein 14 (Sox14), a High mobility group (HMG) box-containing transcription factor related to the mammalian sex determining factor, SRY [75]. dSH3PX1 acts as a binding partner for the non-receptor Cdc-42 associated kinase (ACK) in *Drosophila* [76]. A similar interaction between ACK2 and SH3PX1 (also called SNX9) occurs also in mammals where further studies showed that phosphorylation of SH3PX1 by ACK2 regulates the degradation of EGF receptor [77]. Thus, it is possible that the knockdown of dSH3PX1 by RNAi in *l(2)mbn* cells results in decreased cell death through enhanced EGF receptor-mediated cell survival signaling. Alternatively, the role of dSH3PX1 in cell death may be related to its associations with proteins involved in receptor trafficking and/or cytoskeletal rearrangements [78].

Our *in vitro* and *in vivo* analyses also identified for the first time a pro-death role for the transcription factor Sox14. Previously [24] we determined that of 19 genes tested, just two genes, *Sox14* and *ark*, were independent of E93 regulation in dying larval salivary glands. This previous finding indicates that Sox14 may act in parallel to E93 or may be acting upstream of E93 in the ecdysone induced cell death pathway. Our gene expression analyses reported here (Figure 7E) position Sox14 upstream of E93, and also upstream of rpr and dronc that are known to be regulated by E93 [27]. A recent microarray study conducted during *Drosophila* pupariation further supports this view as *Sox14* was identified as an ecdysone primary-response regulatory gene [79]. Based on comparison of the HMG box region, *Drosophila* Sox14 is most similar to mouse Sox4 and human Sox4, 11 and 22 [75],[80],[81]. Sox proteins regulate multiple downstream targets and are involved in numerous developmental processes. In particular, human Sox 4 has been implicated in both the positive [82],[83] and negative [84] regulation of apoptosis.

Our *in vivo* studies using a *Sox14*-RNAi construct support a pro-death role for *Sox14* during *Drosophila* ecdysone-triggered larval midgut (Figure 6) and salivary gland cell death (Figure 7). During metamorphosis, the larval midgut disintegrates and a new adult gut is formed. These two events overlap and the adult gut encompasses disintegrating larval gut tissue [9]. In *Tub-Sox14-RNAi* animals, adult midgut cells are visible at 4 hrs APF similar to wild type gut, but complete condensation of the larval midgut and complete disintegration of the proventriculus and gastric caecae were inhibited at least up to 12 hrs APF. This observation is similar to what was observed in *BR-C* mutants, but different from *E93* mutants which showed defects in larval midgut compaction but not destruction of the proventriculus and gastric caecae [10]. Thus, the midgut cell death defective phenotype of *Sox14* is again in agreement with our prediction that *Sox14* is acting upstream of E93 and downstream or parallel to BR-C. Our results using both the *Tub-Sox14-RNAi* (tubulin GAL4 driver) and *D59-Sox14-RNAi* (salivary gland GAL4 driver) animals support a role for Sox14 as a positive regulator of salivary gland cell death. Cell death was delayed in *D59-Sox-14 RNAi* animals and was either delayed or inhibited as late as the pharate adult stage in *Tub-Sox14-RNAi* animals. It is possible that the less severe phenotype in the *D59-Sox14-RNAi* animals is due to less efficient RNAi-mediated knockdown of Sox14, a notion that is supported by our observed dose-dependent effects of the *D59-GAL4* driver. Given the predicted function of Sox14 as a transcription factor, it is particularly likely that even reduced amounts could still lead to some wild-type function. It is also possible that Sox14 functions in a partially redundant manner in both the midgut and salivary gland so that even a complete loss of function may lead to only a partial loss or delay in cell death. Null mutants of *Sox14* would be valuable for future testing of these possibilities.

In addition to defects in midgut and salivary gland cell death, we observed tracheal defects in *Tub-Sox14-RNAi* animals, similar to defects observed in mutants of *DHR3* which encodes an ecdysone responsive orphan nuclear receptor [85]. Preliminary examination of *Tub-Sox14-RNAi* pharate adults indicated additional roles for Sox14 in notum and bristle development. Future studies are required to determine the function of Sox14 in these and other tissues. Given its predicted role as a transcriptional regulator and its position in the ecdysone signaling cascade, it is likely that Sox14 will function in various cellular processes.

In summary, we developed an RNAi-based screening system to identify genes that are required for ecdysone-mediated cell death and survival pathways. Our screen identified known and novel components of the ecdysone signaling network that act as pro-death or pro-survival genes. In particular, we have shown that in some cases the function of a gene is dependent on ecdysone, or its mechanism of action is variable depending on the presence or absence of ecdysone. *In vivo* studies of *Sox14* support a role in ecdysone-mediated cell death. Further characterization of the novel genes identified is necessary to elucidate their specific roles and positions in the ecdysone signaling network.

## Materials and Methods

### dsRNA Design and Synthesis

For the initial screen, individual PCR products up to 735 bp in length and containing coding sequences for the transcripts to be knocked-down (Table S1) were generated by RT-PCR using 500 ng of total RNA and Superscript one-step RT-PCR kit with platinum taq (Invitrogen). Each primer used in the RT-PCR contained a 5′ T7 RNA polymerase binding site (TAATACGACTCACTATAGG) followed by sequences specific for the targeted genes (see Table S1). RT-PCR products were isopropanol-precipitated and the entire product from each reaction was used as template for in vitro transcription reactions. In vitro transcription reactions were carried out using either Megascript T7 transcription kit (Ambion) or T7 RiboMax Express RNAi systems (Promega) according to the manufacturer's instruction. dsRNAs synthesized were incubated at 65°C for 30 min followed by slow cooling to room temperature. dsRNAs were ethanol precipitated and resuspended in 50 µl nuclease free water. A 5 µl aliquot of 1/100 dilution was analysed by 1% agarose gel electrophoresis to determine the quality of dsRNA. The dsRNAs were quantitated using a picogreen assay (Invitrogen) and concentrations adjusted to 100 ng/µl with nuclease free water.

For genes of interest identified in our initial screen (for complete list see Table S1), we designed a second non-overlapping dsRNA to confirm the observed phenotype. The German Cancer Research Center (DKFZ) ERNAi search tool (Off-Target Search Tool: http://www.dkfz.de/signaling2/e-rnai/) [86] was used to search the RNAi probes for potential off-target effects using a 19 bp fragment length cut-off . Our final criteria for confirmation of RNAi effects was that for each gene, at least one of its dsRNAs had no predicted off-target effects (i.e. 100% specificity) and a second dsRNA had at least 98% specificity. Of the 20 genes that we confirmed by this method, 19 were represented by two dsRNAs that were completely non-overlapping. One additional gene confirmed by this method, *RpL13A* (*CG1475*), was represented by two dsRNAs that overlapped by 21 bp. Analysis of this 21 bp by the Off-Target Search Tool indicated 0 potential secondary targets.

### Cell Culture and Ecdysone Treatment

*l(2)mbn* cells [42] and S2 cells (Invitrogen) were grown in Schneider's (Invitrogen) medium supplemented with 10% FBS, 50 units/ml penicillin and 50 µg/ml streptomycin (Gibco-BRL) (hereafter referred to as Schneider's medium+10%FBS) in 25-cm^2^ suspension flasks (Sarstedt) at 25°C. All experiments were carried out 3 days after passage and the cells were discarded after 25 passages. 20-Hydroxyecdysone (ecdysone) was obtained from Sigma-Aldrich and resuspended in 95% ethanol at a concentration of 10 mM.

### Quantitative RT-PCR

Three days after passage, cells were adjusted to 1×10^6^ cells/ml in ESF921 serum free media (Expression systems) and 3×10^5^ cells (333 µl) were seeded into each well of a 24 well plate. After one hour incubation, 667 µl of Schneider's medium+10%FBS and ecdysone (10 µM final) (Sigma) was added to yield 1 ml culture in each well. Treated cells were incubated at 25°C for 24, 48, or 72 hours and 1 ml cultures were transferred to RNAse free eppendorf tubes (Ambion) and cells were pelleted at 1000 rpm for 10 min. Cell pellets were lysed in 1 ml Trizol (Invitrogen) and total RNA was extracted according to manufacturer's instructions. Isolated RNA was treated with RNAse free DNAse and 50 ng of total RNA was used in 15 µl QRT-PCR reactions. QRT-PCR reactions were carried out using the one-step SYBR green RT-PCR Reagent kit (Applied Biosystems) on an Applied Biosystems 7900 Sequence Detection System. Expression levels were calculated using the Comparative Cycle Threshold (CT) Method (2^−[delta][delta]Ct^ method; User Bulletin #2, ABI Prism 7700 Sequence Detection System, Applied Biosystems, 2001) with *Drosophila rp49*, a ribosomal housekeeping gene, as the reference for normalization. To determine the fold change in expression levels of known ecdysone signaling genes, cell death related genes and *Sox14* following ecdysone treatment of *l(2)mbn* cells, the CT values were normalized to *rp49* in the same sample (hereafter referred to as normalized CT values) for each gene, and were compared to the normalized CT values for the same gene from untreated control cells. Similarly, for the RNAi experiments, normalized CT values for each gene from ecdysone plus dsRNA-treated cells were compared to the normalized CT values from cells treated with ecdysone plus control human dsRNA, and the knock-down efficiency was calculated.

Knock-down efficiency = 100-[(Fold expression of targeted gene in dsRNA+ecdysone treated cells/Fold expression of targeted gene in ecdysone treated cells) ×100].

### RNA Interference (RNAi) and Cell Viability Assays

For RNAi in *l(2)mbn* cells, a 33 µl volume of ESF921 media containing 3×10^4^ cells was seeded into each well of a 96 well plate for RNAi screens. Into each well, 500 ng–1000 ng of dsRNA in a 5 µl volume was added, and incubated for one hour at room temperature. The untreated control cells received 5 µl of nuclease free water. After one hour incubation, the cells received Schneider's medium+10% FBS containing ecdysone (10 µM final) to yield a final 100 µl volume. Cells were incubated for 72 hours at 25°C and 10 µl of WST-1 reagent (Roche Scientific) was added. A450–A650 readings were taken after overnight incubation using a 96 well spectrophotometer VersaMax (Molecular Devices). A450–A650 readings of experimental samples were always compared to A450–A650 readings from cells treated with human dsRNA to control for any non-specific RNAi effects. RNAi experiments in S2 cells were essentially performed as for *l(2)mbn* cells with the following modifications: 1000–1500 ng of dsRNA in a 5–7.5 µl volume was added. Cells received 1 µM ecdysone overnight and then the ecdysone concentrations were increased to 10 µM final. Cells were incubated for a total of 48 hours (following addition of dsRNA) at 25°C and then 10 µl of WST-1 reagent was added. Assay readings were taken after overnight incubation. All samples were analyzed at least in triplicate.

### Cell Morphology

To quantitate changes in cell shape, indicative of cell differentiation, following ecdysone treatment and RNAi, five images from two biological replicates were captured using an Axiovert 200 fluorescent microscope (Carl Zeiss). The observed number of spindle-shaped and rounded cells were counted manually. Percent spindle-shaped (differentiated) cells was calculated as the Number of spindle-shaped cells/(Number of spindle-shaped cells+ number of rounded cells)*100.

### BrdU (5-Bromo-2′-Deoxyuridine) Cell Proliferation Assay

RNAi experiments were carried out as described above and cell proliferation was determined using the Cell Proliferation BrdU ELISA kit (Roche Scienitific). Cells received BrdU 64 hrs after dsRNA treatment and the cells were incubated for another 24 hrs. Cells were then processed as per manufacturer's instructions and A370–A492 readings were taken using a 96 well spectrophotometer VersaMax (Molecular Devices). A370–A492 readings from experimental RNAi treatments were compared to the reading from cells treated with human dsRNA.

### TUNEL Assay

For the TUNEL assay in vitro, RNAi experiments were carried out as described above except the cells were seeded into each well of 16-well CC2 coated chamber slides (Nunc). After 72 hours of respective treatment, cells received 100 µl of hypotonic solution (75 mM KCl) for 3–5 min. at 25°C. Cells were then fixed with 3∶1 methanol∶acetic acid solution and air dried. Cells were washed with 1XPhosphate buffer saline (Sigma) and processed with TUNEL using the DeadEnd fluorometric tunel system (Promega). Cells were mounted with Slowfade antifade reagent with DAPI (Invitrogen) and viewed using a Zeiss Axioplan 2 microscope. Images were captured using a cooled mono 12 bit camera (Qimaging) and Northern Eclipse image analysis software (Empix Imaging Inc.) and the number of TUNEL positive cells (green) and number of DAPI positive cells (blue nuclear stain) were visually counted. All samples were analysed with at least two biological replicates, and three images from each replicate were taken using a 20× objective for counting the TUNEL and DAPI positive cells. Percent TUNEL positive cells were calculated as (TUNEL positive cells/Total number of cells) ×100.

For the TUNEL assay in tissue, dissected salivary glands were first fixed with 4% paraformaldehyde and then permeabilized with 1% TritonX-100. The TUNEL procedure was performed using the DeadEnd fluorometric tunel system (Promega) and salivary glands were mounted in Slowfade antifade with DAPI (Invitrogen) and viewed as described above.

### Sox14 Expression Constructs and Transfection

Plasmids were constructed using the GATEWAY system (Invitrogen) as follows: A full length cDNA was first generated as an RT-PCR product from total *Drosophila* RNA using Superscript III and platinum Pfx polymerase (Invitrogen) with the gene-specific primers GAAGACGCGTCAAAGCATTTATTCTCGCGTTT and TATGGTACCTAGTGCACAC TCACACTCG (underlined portions represent sequence added to create restriction enzyme sites for subsequent cloning). PCR products containing the *Sox14* ORF flanked by AttB1and AttB2 sequences were amplified from the full length cDNA using platinum Pfx polymerase (Invitrogen). The primers GGGGACAAGTTTGTACAAAAAAGCAGGCTTC ATAGCTAAGCCCAACCAGGC and GGGGACCACTTTGTACAAGAAAGCTGGGTC TCACATTTTATAATAACTTGCAAACTCG were used to amplify a product suitable for an N-terminal fusion construct and the primers GGGGACAAGTTTGTACAAAAAAGCA GGCTTCACCATGATAGCTAAGCCCAACCAG and GGGGACCACTTTGTACAAGAAA GCTGGGTCCATTTTATAATAACTTGCAAACTCGTATT were used to generate a product for the C-terminal fusion construct. (*Sox14*-specific sequences are underlined.) The PCR products were cloned into the entry clone, pDONR221, (Invitrogen) which contains AttP sites. These entry clones were then used to shuttle the protein-coding region of the genes into the GATEWAY expression vectors pAFW or pAWF, containing either N-or C-terminal FLAG respectively (Drosophila Genomics Resource Center), as required.

Sox14-FLAG expression constructs were used for overexpression studies in *l(2)mbn* cells. *l(2)mbn* cells were grown as described above. For transfection experiments, 1 µg of plasmid DNA and 10 µl of Cellfectin (Invitrogen) were combined in 200 µl of serum-free Grace medium (Invitrogen) for 30 min. Immediately prior to transfection, 3×10^6^ cells in 800 µl of Grace medium were prepared and incubated with the transfection medium (a total of 1 ml culture) overnight in a 24 well suspension culture plate (Sarstedt). Cells were equally spilt into two wells (500 µl each), and each well received 1 ml of Schneider+10%FBS medium. Cells were incubated for up to 96 hrs. At 48 and 72 hrs after transfection, 100 µl of cells were plated into a 96 well plate, 10 µl of WST-1 reagent was added, and absorbance readings were taken after overnight incubation. WST-1 readings of cells transfected with Sox14 expression constructs were compared to the cells transfected with empty vector (negative control). Transfected cells were monitored for the presence of apoptotic bodies up to 96 hrs after transfection. Samples were analyzed in triplicate, with two biological replicates of each construct.

### *Sox14-RNAi* Lines

A *UAS-Sox14-RNAi Drosophila* line was obtained from the Vienna Drosophila RNAi Center. Heterozygous animals containing the *Sox14-RNAi* construct, balanced over *TM6B*, *Tb^1^* were crossed to a stock carrying the *Tubulin-GAL4* driver (w* ; *Tub-GAL4/TM6B*, *Tb^1^* , derived from *TubP-GAL4^LL7^* (Bloomington stock centre)) to drive the expression of *Sox14-RNAi in vivo*. The phenotype of the resulting F1 non-tubby progeny (*Sox14-RNAi/Tub-GAL4*; designated *Tub-Sox14-RNAi*) was compared to control animals (*Tub-GAL4/+*) designated as wild-type (wt). The knock-down efficiency of *Sox14* was determined by comparing transcript levels in *Sox14-RNAi/Tub-GAL4* animals to the wt +/*Tub-GAL4* animals using QRT-PCR as described above. For salivary gland-specific *Sox14*-*RNAi* studies, animals containing *UAS-Sox14-RNAi* were crossed to a strain containing the D59 salivary gland driver [87] and a strain containing two copies of salivary gland driver and one copy of *UAS-Sox14-RNAi* was established (*D59-GAL4/D59-GAL4*; *Sox14-RNAi/TM6B*; designated *D59-Sox14-RNAi*). Control animals were *D59-GAL4/D59-GAL4*; *MKRS/TM6B*.

### Phalloidin Staining of Retinae

Retinae were dissected from *Tub-Sox14-RNAi* animals following salivary gland dissection. Retinae were fixed with 4% paraformaldehye for 20 minutes and permeabilized with 1% TritonX-100. Phalloidin-Rhodamine (Invitrogen) was used to stain and reveal outline of cells. Retinae were mounted in Slowfade antifade with DAPI and viewed on a Zeiss Axioplan 2 microscope. Ommatidial organization, cell number and apical profile were used to assess developmental age [59].

### Statistical Analyses

Probability p-values were calculated with Student's t-test using two-tailed distribution and two-sample equal variance.

### Online Supplemental Material

Table S1 provides a complete list of genes targeted by RNAi along with their primer sequences, amplicon lengths, cell viability results and p values. Table S2 includes a comparison of WST-1 assay results in *l(2)mbn* and S2 cells for the identified candidate pro-death and pro-survival genes. Figure S1 shows the cell viability effects of Atg gene RNAi in the absence of ecdysone and Figure S2 shows results of the BrdU cell proliferation assay.

## Supporting Information

Table S1 List of names, primer sequences, and viability results for genes targeted by RNAi.
(0.20 MB XLS)

Table S2 Comparison of RNAi effects on cell viability and ecdysone dependency in *l(2)mbn* and S2 cells.
(0.07 MB DOC)

Figure S1 RNAi of some Atg genes shows reduced viability in the absence of ecdysone.
(0.55 MB TIF)

Figure S2 Analysis of cell proliferation by the BrdU incorporation assay in dsRNA-treated *l(2)mbn* cells.
(1.88 MB TIF)
