# Supplementary material for: Steroid Hormone Control of Cell Death and Cell Survival: Molecular Insights Using RNAi
Source: PLoS Genet. 2009 Feb 13;5(2):e1000379. doi: 10.1371/journal.pgen.1000379 (PMC2632862; doi:10.1371/journal.pgen.1000379)
Supplement: Table S2 — Comparison of RNAi effects on cell viability and ecdysone dependency in l(2)mbn and S2 cells. (0.07 MB DOC) [file pgen.1000379.s002.doc]

**Supplementary Table 2**.

Comparison of RNAi effects on cell viability and ecdysone dependency in *l(2)mbn* and S2 cells

| **Gene symbol and CG number** | **WST-1 assay in**  **l(2) mbn: dsRNA+ecdysone (p≤0.05)** | **Ecdysone dependency in l(2)mbn** | **WST-1 assay in**  **S2 cells: dsRNA+ecdysone** | **Ecdysone dependency in S2** |
| --- | --- | --- | --- | --- |
| **Control Pro-death** |  |  |  |  |
| EcR | 6.9E-05 | dependent | 8.6E-08 | dependent |
| Hid | 2.8E-03 | dependent | 7.2E-02 | NE |
| BR-C | 1.6E-02 | dependent | 1.0E-04 | dependent |
| Nc (dronc) | 1.9E-02 | dependent | 3.1E-06 | dependent |
| reaper | 1.0E-02 | dependent | 1.0E-03 | dependent |
| **Candidate pro-death** |  |  |  |  |
| RpL13A | 6.3E-05 | dependent | 4.6E-02 | independent* |
| Sox14 | 6.7E-04 | dependent | 2.2E-01 | NE |
| RpS6 | 1.5E-03 | dependent | 3.7E-02 | independent* |
| RpLP1 | 3.2E-03 | dependent | 2.7E-02 | independent* |
| RpS5 | 5.6E-03 | dependent | 3.9E-02 | independent* |
| SH3PX1 | 1.6E-02 | independent | 1.2E-03 | independent |
| RpL37 | 5.4E-02 | dependent | 2.0E-03 | independent* |
| **Control Pro-survival** |  |  |  |  |
| th(diap-1) | 8.8E-04 | independent | 1.1E-05 | independent |
| E75 | 7.0E-04 | dependent | 7.4E-04 | dependent |
| **Pro-survival** |  |  |  |  |
| sin3A | 3.1E-04 | independent | 7.0E-03 | independent |
| S6K | 6.4E-04 | independent | 1.2E-01 | NE |
| Rpn2 | 8.0E-04 | independent | 9.6E-03 | independent |
| Pros26.4 | 8.8E-04 | independent | 3.0E-04 | independent |
| Ras85D | 2.0E-03 | dependent | 5.8E-01 | NE |
| Smr | 2.1E-03 | independent | 9.3E-03 | independent |
| Vps32 | 3.2E-03 | independent | 2.3E-02 | independent |
| Tbp-1 | 3.6E-03 | independent | 1.7E-04 | independent |
| Tor | 6.4E-03 | independent | 8.1E-02 | NE |
| CG33087 | 6.5E-03 | dependent | 6.6E-01 | NE |
| Indy | 7.1E-03 | independent | 4.0E-02 | independent |
| Kap-α3 | 7.4E-03 | independent | 3.7E-03 | independent |
| CG7466 | 8.5E-03 | independent | 5.3E-01 | NE |
| Cp1 | 1.0E-02 | dependent | 2.5E-02 | dependent |
| CG32016 | 2.0E-02 | dependent | 4.3E-02 | dependent |
| HmgD | 2.1E-02 | independent | 5.5E-01 | NE |
| CG13784 | 5.8E-03 | dependent | 2.4E-01 | NE |
| CG15239 | 3.6E-02 | independent | 3.4E-02 | independent |

Gene symbols, and CG numbers (column 1), are from Flybase [58]. Columns 2 and 3 from Table 1 are included here for comparison. P-values in columns 2 and 4 were calculated by comparing the WST-1 reading (A450-A650) of RNAi of the gene of interest to the WST-1 reading of RNAi of the human negative control (NM_138278). Ecdysone dependency in columns 3 and 5 for l(2)mbn and S2 cells, respectively, was based on the observation that the observed viability effects of RNAi depended on the presence of ecdysone. Cell viability phenotypes in *l(2)mbn* cells and S2 cells were similar in most cases. However, in S2 cells, dsRNA treatment of ribosomal genes (indicated by *) showed a pro-survival effect both with and without ecdysone treatment. NE in column 5 indicates that similar trends were observed between both cell types but the effects were not statistically significant (p>0.05).
