# Supplementary figures and images for: Steroid Hormone Control of Cell Death and Cell Survival: Molecular Insights Using RNAi
Source: PLoS Genet. 2009 Feb 13;5(2):e1000379. doi: 10.1371/journal.pgen.1000379 (PMC2632862; doi:10.1371/journal.pgen.1000379)

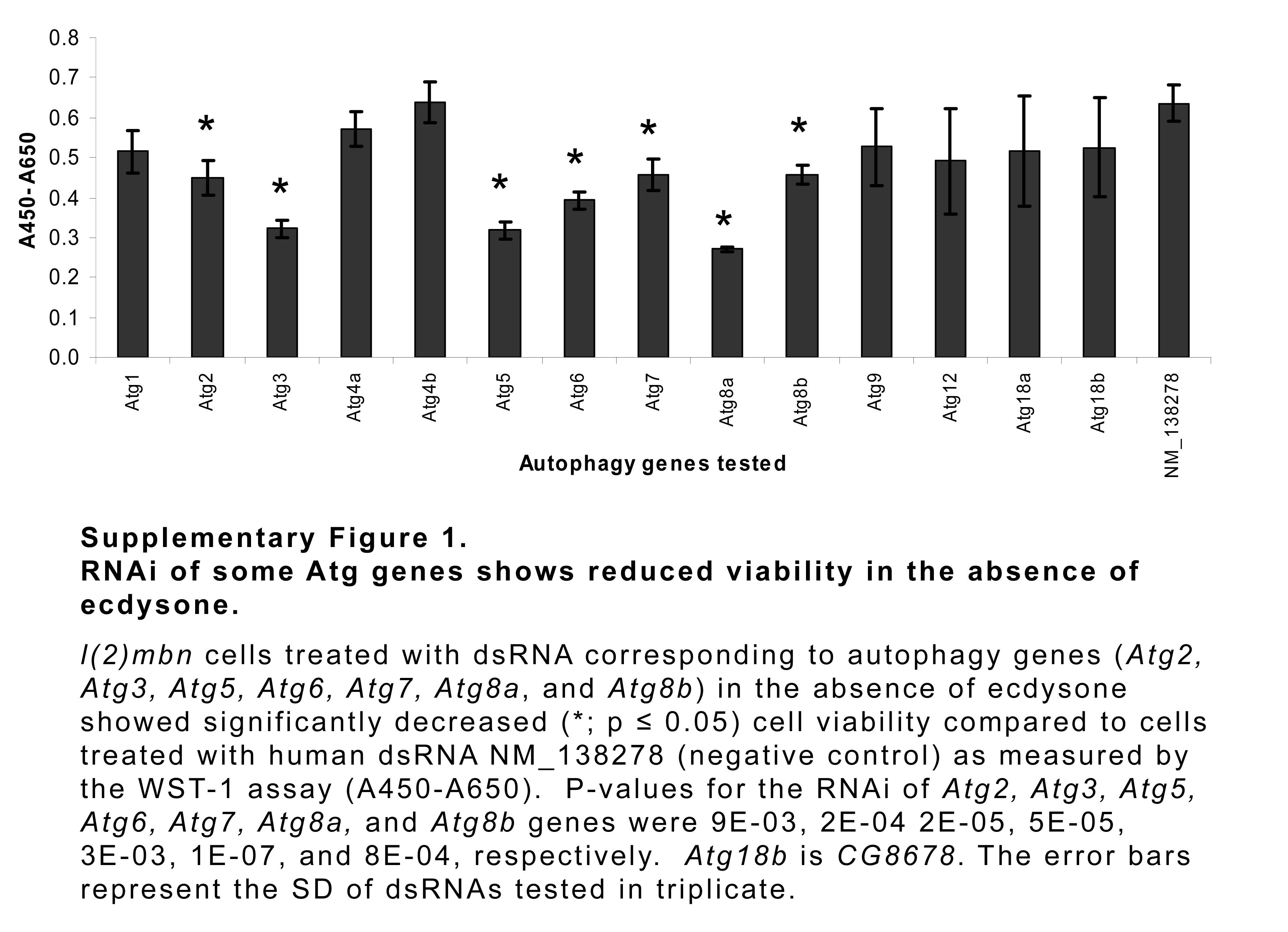

Supplement: Figure S1 — RNAi of some Atg genes shows reduced viability in the absence of ecdysone. (0.55 MB TIF) [file pgen.1000379.s003.tif]

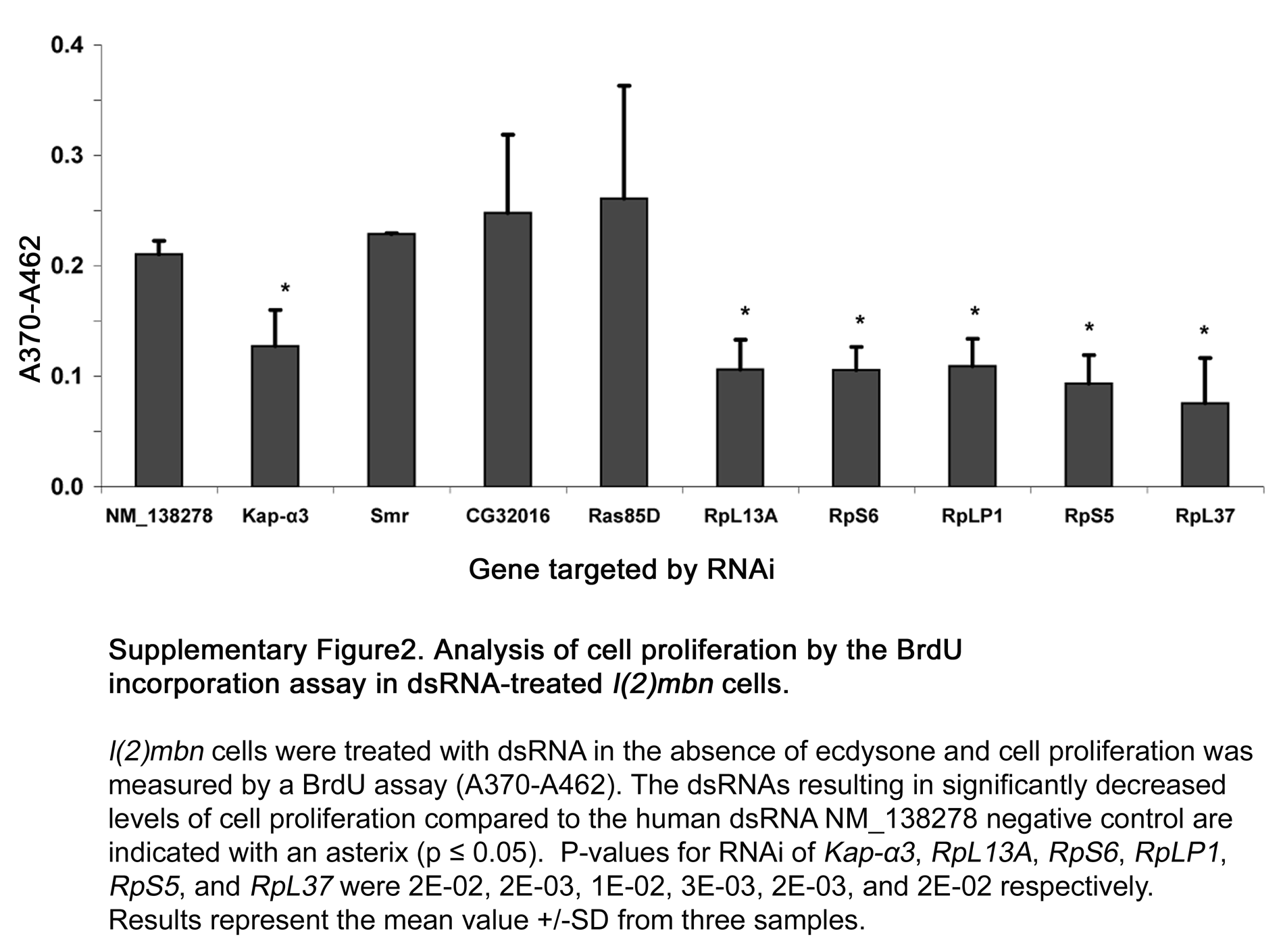

Supplement: Figure S2 — Analysis of cell proliferation by the BrdU incorporation assay in dsRNA-treated l(2)mbn cells. (1.88 MB TIF) [file pgen.1000379.s004.tif]
